# Cognitive function characteristics and influencing factors in patients with depressive disorders: a 5-year follow-up report based on the epidemiological survey of mental disorders in Shandong Province, China

**DOI:** 10.3389/fpsyt.2024.1434659

**Published:** 2024-10-15

**Authors:** Ziang Lin, Qian Wang, Xiaojing Li, Can Wang, Ruzhan Wang, Chenghui Wang, Hao Ding, Liju Qian, Xiaona Wan, Xue Tian, Zongyin Hou, Fengjie Liu, Jindong Liu, Jingxuan Zhang, Xiaojing Cheng

**Affiliations:** ^1^ Department of Psychiatry, School of Mental Health, Jining Medical University, Jining, China; ^2^ Shandong Province Hospital of Occupational Diseases, Jinan, China; ^3^ Shandong Mental Health Center, Jinan, Shandong, China; ^4^ Weifang Mental Health Center, Weifang, Shandong, China; ^5^ Zibo Mental Health Center, Zibo, Shandong, China; ^6^ Daizhuang Hospital, Jining, Shandong, China; ^7^ Qingdao Mental Health Center, Qingdao, Shandong, China; ^8^ Linyi Mental Health Center, Linyi, Shandong, China; ^9^ Zaozhuang Mental Health Center, Zaozhuang, Shandong, China; ^10^ The Fourth People’s Hospital of Liaocheng, Liaocheng, Shandong, China; ^11^ The Third People’s Hospital of Heze, Heze, Shandong, China; ^12^ Shandong Academy of Occupational Health and Occupational Medicine, Jinan, Shandong, China

**Keywords:** depressive disorder, cognitive function, case-control study, epidemiological survey, Shandong province

## Abstract

**Objective:**

Cognitive impairment represents a notable feature of depressive disorders. Comprehending its characteristics and influencing factors is vital for patient rehabilitation.

**Methods:**

This study is based on the 2015 Shandong Province epidemiological survey of mental disorders, from which 871 individuals meeting DSM-IV criteria for depressive disorders were selected as the research group. Using 1:1:1 matching by sex, age, and residence, we randomly selected 825 individuals with no DSM-IV diagnosis but positive on GHQ-12 and additional risk assessments as the elevated risk control group, and 825 with negative screenings as the minimal risk control group. In 2020, a follow-up survey was conducted, resulting in a final analysis of 1,855 cases. The survey included demographic data, various clinical information, and a series of screening and questionnaire assessments.

**Results:**

The current depressive group scored lower on the MoCA than the non-depressive group (*t*=8.86, *P*<0.01). The research group scored lower on the MoCA than the elevated and minimal risk group (*F*=11.98, *P*<0.01). The depression-unremitted group scored lower than the depression-remitted group (*t*=6.44, *P*<0.01). The Analysis indicated that males, with better life quality, poor early psychological status and, and longer education were associated with higher MoCA scores. Conversely, older age, rural residency, employment, current depression, and poor marital were associated with lower MoCA scores.

**Conclusion:**

Individuals with depression commonly suffer cognitive impairment, which tends to partially improve as depression remits. Individual cognitive function is influenced by early psychological health, depressive status, quality of life, age, sex, educational level, residence, occupational, and marital status.

## 1 Introduction

Depressive Disorder is characterized primarily by significant and persistent low mood, with high incidence, high recurrence rates, and high disability rates. Currently, there are more than 300 million individuals with Depressive Disorder worldwide, constituting 4.4% of the global population (1), making it a significant health issue affecting the well-being of the population.

In addition to affective symptoms, cognitive dysfunction is also a common clinical manifestation among individuals with depression. Cognitive dysfunction is a group of clinical syndromes characterized by a decline in cognitive function resulting in impaired cognitive activities and social functioning (2). Studies have found that nearly ninety percent of patients with depressive disorders experience cognitive dysfunction during depressive episodes, and 39%-44% of patients continue to experience it even after the symptoms of depression have subsided (3). Patients with severe depressive disorder exhibit significant impairments in multiple cognitive domains, including attention, executive function, language, and spatial cognition (4), and the more frequent the depressive episodes, the more severe the cognitive dysfunction (5). Conversely, impairments in cognitive functions can also affect emotional regulation abilities, making patients more prone to negative emotions. This creates a vicious cycle that affects the recovery and recurrence of depressive disorder (6). Although there has been significant progress in the research and improvement of cognitive dysfunction in depressive disorders over time, the characteristics of cognitive functions and their influencing factors remain a highly debated research focus.

While numerous studies abroad have explored cognitive impairments in patients with depressive disorders, large-scale epidemiological research in this area is limited in China, and no consistent conclusions have been reached. Shandong Province, located along China’s eastern coast, is a significant province in terms of population, economy, and agriculture. This study utilized samples from Shandong Province’s fourth epidemiological survey of mental disorders (2015). A follow-up study was conducted after 5 years (2020) with patients with depressive disorders and elevated and minimal risk groups. Diagnostic and scale assessments were completed at two time points to understand the impact of past and current depression, as well as various demographic and social factors, on cognitive function. The study aimed to explore the characteristics and influencing factors of cognitive impairment in depressive disorders, providing insights for the prevention and treatment of cognitive dysfunction in patients.

## 2 Methods

### 2.1 Study design and participants

This study is based on the 2015 epidemiological survey of mental disorders in Shandong Province, China (7). For this follow-up study, 871 patients were randomly selected from the 1,237 individuals identified in the aforementioned survey as meeting the Diagnostic and Statistical Manual of Mental Disorders, Fourth Edition (DSM-IV) criteria for major depressive disorder, dysthymic disorder, or not otherwise specified depressive disorder. These patients constituted the research group. Using a 1:1:1 matching principle with the research group based on gender, age group (± 5 years of the research group), and residential area (same village or street), 825 individuals were randomly selected as the elevated risk control group from the 2015 survey participants who were assessed as having no mental disorders according to the DSM-IV, but who tested positive on the General Health Questionnaire (GHQ-12) and additional risk assessment questions. Additionally, 825 individuals were randomly selected from the 2015 survey participants who were assessed as having no mental disorders according to the DSM-IV, and who tested negative on the GHQ-12 and related risk factor screening, to serve as the minimal risk control group. Due to a shortage of members in the elevated and minimal risk groups meeting the matching criteria and principles to achieve 871 cases, the numbers across the three groups are not identical.

From November 2020 to March 2021, a follow-up survey was conducted on the aforementioned three groups, comprising a total of 2,521 individuals. Of these, 2,122 individuals (84.17%) completed the follow-up, while 267 individuals did not complete the survey due to refusal, death, or other reasons. Ultimately, 1,855 participants (73.58%) were included in the analysis: 518 in the research group, 635 in the elevated risk group, and 702 in the minimal risk group. The participants’ ages ranged from 27 to 98 years, with an average age of 63.89 ± 11.59 years. Among them, 588 were male (31.7%) and 1,267 were female (68.3%). In the research group, the average age was 64.47 ± 11.10 years, with 151 males (29.2%) and 367 females (70.8%); in the elevated risk group, the average age was 63.68 ± 11.89 years, with 216 males (34.0%) and 419 females (66.0%); in the minimal risk group, the average age was 63.65 ± 11.67 years, with 221 males (31.5%) and 481 females (68.5%). There were no significant differences in gender or age among the three groups (*P* = 0.21, *P* = 0.40).

### 2.2 Ethics statement

This study was approved by the Ethics Committee of the Shandong Mental Health Center, with the ethical approval number being (2019) Ethical Review No. (R11). All participants or their guardians provided written informed consent.

### 2.3 Survey tools

#### 2.3.1 Basic data collection questionnaire

It is a self-designed survey instrument used to gather basic demographic data from respondents, such as age, gender, living arrangements, level of education, marital status, sources of medical care, etc.

#### 2.3.2 Diagnostic criteria and instruments

The investigation adopted the American Psychiatric Association’s revised DSM-IV as the diagnostic criteria for depressive disorders (8). The Chinese version of the Structured Clinical Interview for DSM-IV Axis I Disorders, Patient Edition (SCID-I/P) (9), was used as the diagnostic tool to assess the mental status of the study population. Its consistency has been validated, with Kappa values ranging from 0.92 to 0.94 (10).

#### 2.3.3 Screening tools

In this study, the GHQ-12 and additional risk assessment questions were used as screening tools (11). The GHQ-12 is a commonly utilized tool for assessing mental health status, consisting of 12 questions with a total score ranging from 0 to 12. Additionally, this study incorporated 9 new risk assessment questions to enhance sensitivity in screening (12), which are: 1. Self-perceived poor physical health in the past month; 2. Self-perceived poor mental health in the past month; 3. Frequent and uncontrollable recurrent thoughts or actions in the past month; 4. Frequent restriction of activities due to specific fears in the past month; 5. Frequent feelings of tension or anxiety in the past six months; 6. Regular alcohol consumption or at least two instances of drunkenness in the past year; 7. Seeking medical advice for mental or psychological issues; 8. Receiving inpatient treatment for mental or psychological issues; 9. Hospitalization or frequent visits to hospitals due to physical illnesses in the past three years. The scale demonstrates good reliability and validity in the Chinese population, with a Cronbach’s α value of 0.79 (13). A total GHQ-12 score of ≥1 or the presence of one or more risk factors was considered a positive screening result.

#### 2.3.4 Montreal cognitive assessment

The scale was developed by Canadian scholars Nasreddine et al. and is widely used for screening cognitive impairment. It covers seven major cognitive domains: 1. Visuospatial and Executive Function: Assessed through the Trail Making Test Part B, cube drawing, and clock drawing test, evaluating visual-spatial abilities, planning, and executive functions respectively; 2. Naming Ability: Evaluated by recognizing and naming three animal pictures; 3. Attention: Measured using digit span tests, digit 1 test, and subtraction tasks, which assess attention, calculation ability, and working memory; 4. Language Ability: Assessed through repetition and verbal fluency tests; 5. Abstract Thinking: Evaluated by similarity tests; 6. Delayed Recall: Assessed through delayed recall tests to evaluate delayed memory and learning ability; 7. Orientation: Measured by asking about the date and location (14). The reliability and validity of this scale have been validated in the Chinese population, with a Cronbach’s α value of 0.82 (15). The total score ranges from 0 to 30, with scores ≥ 26 considered normal. Higher scores indicate better cognitive function.

#### 2.3.5 Global pain scale

The scale, created by Gentile, is a comprehensive and integrative self-assessment tool for pain, consisting of 20 items across four dimensions: pain intensity, emotional impact, clinical manifestations, and daily activities. The reliability and validity of this scale have been verified, with a Cronbach’s α value of 0.95 (16). A higher total score on the scale reflects a more pronounced impact of pain.

#### 2.3.6 Pittsburgh sleep quality index

The instrument, devised by Buysse et al., is used to assess the sleep quality of participants over the past month (17). The Chinese adaptation of the PSQI has demonstrated good reliability and validity among the Chinese population, with a Cronbach’s α value of 0.84 (18). The scale consists of 19 self-rated items and 5 other-rated items, with total scores ranging from 0 to 21. Higher scores indicate poorer sleep quality.

#### 2.3.7 Quality of life questionnaire

The scale was developed by Wang Zhiqing et al. based on the characteristics of the Chinese population, primarily for assessing participants’ quality of life (19). It has demonstrated good reliability, validity, and usability among the Chinese population (20), with some scholars reporting a Cronbach’s α value of 0.81 (21). The scale consists of 6 items with a total score ranging from 0 to 100, where higher scores indicate better quality of life.

#### 2.3.8 Clinical data survey form

A self-designed questionnaire was used to collect disease-related information from patients with mental disorders over a 5-year period (from January 2016 to December 2020), including data on the course of depression, use of various psychiatric medications, presence of physical and other mental illnesses, hospitalization due to mental illness, suicidal behavior, and other clinically relevant information such as incidents involving harm or violence.

### 2.4 Site survey

**Figure 1:**
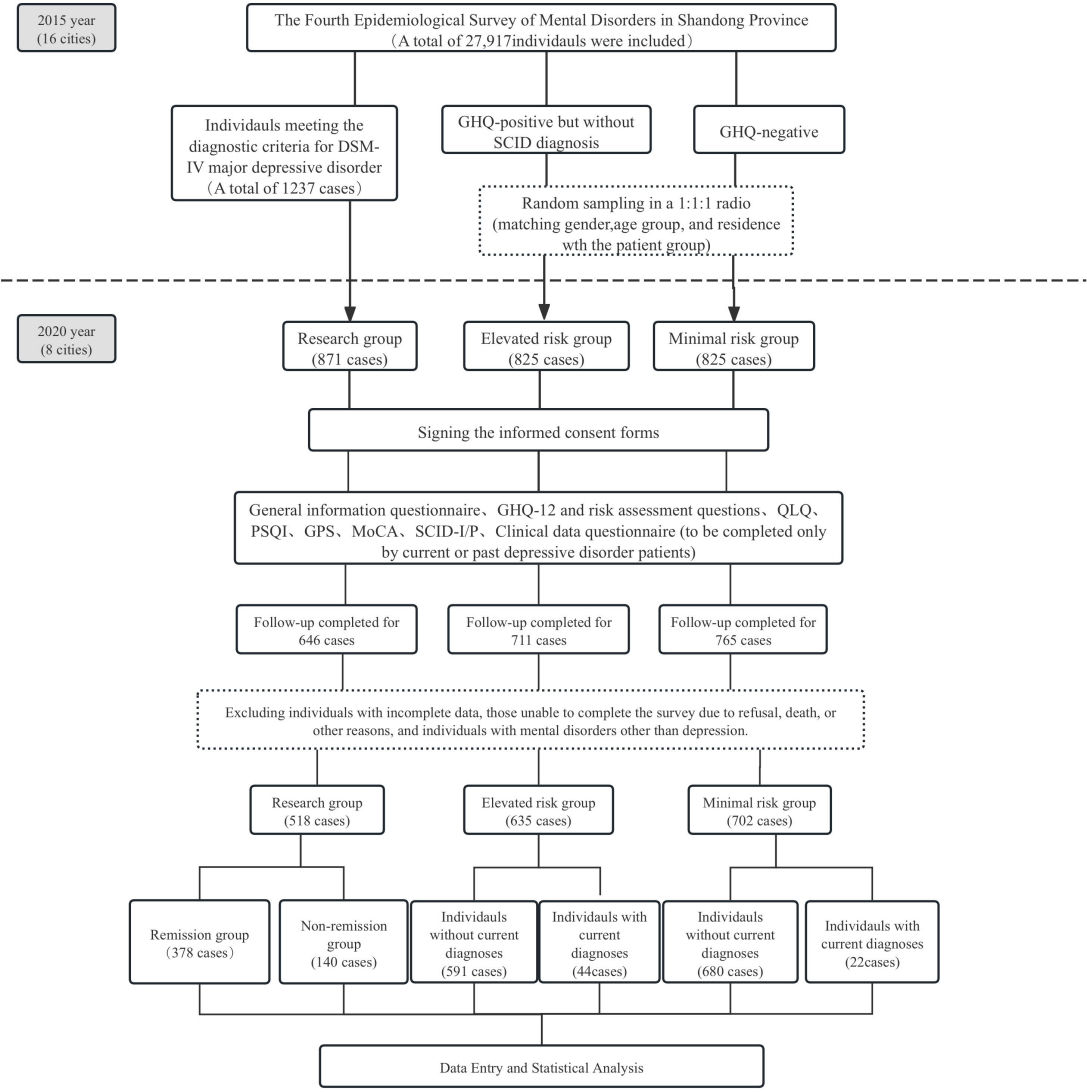
Research flowchart.

Prior to the 2020 follow-up survey, in accordance with the study design, data on the follow-up subjects, including family and general information, diagnosis, and treatment details, were extracted from the 2015 Shandong Province Mental Disorders Epidemiological Survey Database to establish a study cohort database. A total of 56 psychiatrists from 8 cities, who had participated in the 2015 epidemiological survey, were selected as core team members, each responsible for the on-site survey work in their respective cities. The investigators, assisted by local guides, conducted household surveys, obtained informed consent from the follow-up subjects, and then sequentially administered the general information questionnaire, GHQ-12 and related questions, QLQ, PSQI, GPS, and MoCA assessments. Subsequently, the SCID-I/P diagnostic assessment was performed. For patients diagnosed with current (within the past month) or past depressive disorders (including major depressive disorder, dysthymia, and unspecified depressive disorder), a clinical data questionnaire was completed. For individuals without any current or past diagnosis of depressive disorders, the investigation has been completed, and no further information will be collected. For those unable to complete the follow-up survey due to physical illness, refusal to participate, death, etc., the surveyors were required to register their specific circumstances. For patients whose current diagnosis is inconsistent with their 2015 epidemiological survey diagnosis or for newly identified cases that are difficult to diagnose, the diagnosis will be reviewed by quality control personnel from the Shandong Mental Health Center. The research flowchart is shown in **Figure 1**.

### 2.5 Quality control

Psychiatrists from various municipal psychiatric hospitals were selected as field investigators. Before the survey commenced, the Shandong Provincial Mental Health Center conducted comprehensive training on the use of survey instruments, survey procedures, research content, and other relevant topics. Following the training, all survey personnel underwent evaluations and consistency checks related to the survey instruments. The ICC scores for various assessment tools ranged from 0.86 to 0.95, and the diagnostic consistency Kappa values were all above 0.98. The entire survey process was recorded with the respondents’ consent. Additionally, the Shandong Provincial Mental Health Center appointed physicians with intermediate and advanced professional titles to oversee on-site supervision, quality control, and data analysis, all of whom participated in the training sessions.

### 2.6 Statistical analysis

Data entry and analysis were performed using SPSS 26.0 software. Given the approximate normal distribution of the dependent variable MoCA scores, a T-test was employed to compare the scoring of MoCA between the currently depressed and the non-depressed group, as well as between the depression-remitted and depression-unremitted group. This comparison aimed to understand the cognitive function differences among individuals with depression and the general population, as well as within individuals with depressive disorders across the stages of depression remitted and non-remitted. An ANOVA was performed to assess the variance in MoCA scores across the depression group as well as the Elevated and Minimal Risk groups, utilizing the conservative Bonferroni correction method for pairwise comparisons to control the type I error rate due to multiple comparisons, thus understanding cognitive function differences among various depression risk groups. Multiple linear regression analyses were performed to investigate the factors that impact cognitive function in the study population and depressed population. MoCA scores were used as the dependent variable, while demographic factors, disease-related information, and GHQ-12, QLQ, PSQI scores from both 2015 and 2020, as well as GPS scores from 2020, were used as independent variables. Categorical variables were dummy coded, and the variance inflation factor (VIF) was applied to assess multicollinearity among the independent variables. Measurement data are presented as mean ± standard deviation ( $\bar{x}\pm s$ ). Hypothesis tests were two-sided, with a significance level set at α = 0.05. Differences were considered statistically significant if *P* < 0.05.

## 3 Results

### 3.1 Cognitive function analysis of the research population

During the 2020 follow-up assessment, analyses of SCID-I/P diagnostic outcomes revealed that 206 subjects were diagnosed with depressive disorders in the prior month and were classified as the “current depressive group”. The “non-current depressive group” comprised the remaining 1,649 individuals. A comparison of MoCA scale scores between these two groups revealed that the current depressive group had lower MoCA total scores and individual factor scores, demonstrating statistically significant differences (*P* < 0.01) across all analyses. Refer to **Table 1** for a detailed breakdown.

**Table 1:** Comparison of MoCA scale scores: non-current vs. current depressive groups ( $\bar{x}\pm s$ ).

MoCA factors	Non-current depressive(*n*=1649)	Current depressive(*n*=206)	*t*	*P*
Total scores	17.13 ± 6.74	12.73 ± 6.45	8.86	< 0.01
Visuospatial and Executive Function	1.83 ± 1.69	1.13 ± 1.47	6.37	< 0.01
Naming Ability	2.04 ± 1.04	1.55 ± 1.16	5.77	< 0.01
Attention	4.06 ± 1.87	3.10 ± 2.06	6.38	< 0.01
Language Ability	1.45 ± 1.03	0.85 ± 0.85	9.40	< 0.01
Abstract Thinking	0.64 ± 0.81	0.29 ± 0.59	7.54	< 0.01
Delayed Recall	1.18 ± 1.60	0.56 ± 1.14	6.99	< 0.01
Orientation	4.97 ± 1.41	4.27 ± 1.81	5.35	< 0.01

The comparison of MoCA Scale Scores between the Research Group and Elevated/Minimal Risk Control Groups exhibited notable statistical variances in both MoCA total score and individual factor scores among the three groups (*P* < 0.05). Subsequent pairwise analyses unveiled that the MoCA total score and subscale scores within the research group were markedly inferior to those within the minimal risk group (*P* < 0.05). Moreover, the MoCA total score, visuospatial and executive function, attention, language ability, and abstract thinking factor scores within the research group were notably lower than those within the elevated risk group (*P* < 0.05). For comprehensive data, refer to **Table 2**.

**Table 2:** Comparison of MoCA scale scores in the research group vs. elevated/minimal risk groups ( $\bar{x}\pm s$ ).

MoCA factors	Research group (*n*=518)	elevated risk group (*n*=635)	minimal risk group (*n*=702)	*F*	*P*
Total scores	15.45 ± 6.94	16.81 ± 6.91	17.36 ± 6.62^*^	11.98	< 0.01
Visuospatial and Executive Function	1.50 ± 1.63	1.81 ± 1.70^*^	1.90 ± 1.69^*^	8.97	< 0.01
Naming Ability	1.88 ± 1.11	2.01 ± 1.07	2.05 ± 1.04^*^	3.52	0.03
Attention	3.68 ± 2.02	4.00 ± 1.88^*^	4.11 ± 1.85^*^	7.25	< 0.01
Language Ability	1.22 ± 1.03	1.40 ± 1.02^*^	1.49 ± 1.02^*^	10.50	< 0.01
Abstract Thinking	0.50 ± 0.75	0.62 ± 0.80^*^	0.66 ± 0.81^*^	6.87	< 0.01
Delayed Recall	0.97 ± 1.46	1.14 ± 1.60	1.19 ± 1.61^*^	3.52	0.03
Orientation	4.73 ± 1.61	4.88 ± 1.47	5.03 ± 1.36^*^	6.01	< 0.01

*P < 0.01 compared to the research group.

A multiple linear regression analysis was conducted with the total MoCA score of the study population as the dependent variable. Independent variables included gender, age, residence area, years of education, marital status, living arrangement, employment status, source of medical care, current depression status, presence of other mental illnesses, presence of physical illnesses, and total scores for GHQ-12, QLQ, and PSQI from both 2015 and 2020, as well as GPS scores from 2020. The results indicated that gender, age, employment status, years of education, marital status, residence area, current depression status, QLQ total scores from 2015 and 2020, and GHQ-12 total score from 2015 were factors affecting cognitive function (*P* < 0.05). Specifically, males, individuals with better quality of life, poorer early psychological conditions, and higher educational levels exhibited better cognitive function. Conversely, older age, rural residence, employment, current depression, and being unmarried/divorced/widowed were associated with poorer cognitive function. After standardizing the partial regression coefficients for each influencing factor, years of education and age were identified as the primary and secondary factors affecting cognitive function in the study population, respectively. For detailed results, please consult **Table 3**.

**Table 3:** Factors influencing cognitive function in the study population: multivariate linear regression.

Factors	*β*	*SE*	*B*	*t*	*P*	*VIF*
Male ^a^	1.17	0.25	0.08	4.70	<0.01	1.23
Age	-0.17	0.01	-0.29	-15.67	<0.01	1.50
Rural residence ^b^	-1.28	0.25	-0.09	-5.15	<0.01	1.21
Unmarried/divorced/widowed ^c^	-0.67	0.34	-0.04	-1.97	0.05	1.68
Employment ^d^	-0.59	0.29	-0.03	-2.04	0.04	1.21
Years of education	0.84	0.03	0.48	25.44	<0.01	1.54
Current depression ^e^	-0.92	0.44	-0.04	-2.11	0.04	1.70
QLQ total score (2020)	0.03	0.01	0.04	2.02	0.04	1.80
QLQ total score (2015)	0.04	0.01	0.07	3.22	<0.01	1.73
GHQ-12 total score (2015)	0.13	0.05	0.05	2.34	0.02	1.56

R^2^ = 0.57, adjusted R^2^ = 0.57, F=135.24, P<0.01, Durbin-Watson=1.97.

a Female as a reference.

b Urban residence as a reference.

c Married as a reference.

d Unemployed as a reference.

e Not currently depressed as a reference.

### 3.2 Cognitive function analysis of the depressed population

Following the 2020 follow-up survey findings, 378 participants in the research group, evaluated through SCID-I/P as having no depressive disorders and no other mental disorder diagnoses, were defined as the “ Depression-Remitted Group”. Meanwhile, 140 participants retaining diagnoses of depressive disorder were defined as the “Depression-Unremitted Group”. Upon comparing the MoCA scale scores between the two groups, revealed that the MoCA total score and subscale scores in the Depression-Unremitted Group were markedly lower than those in the Depression-Remitted Group (*P <*0.01). Refer to **Table 4** for a comprehensive breakdown of the findings.

**Table 4:** Comparison of MoCA scale scores: depression-remitted group vs. depression-unremitted group ( $\bar{x}\pm s$ ).

MoCA scores	Unremitted Group (*n*=140)	Remitted Group (*n*=375)	*t*	*P*
Total scores	12.35 ± 6.54	16.60 ± 6.73	6.44	<0.01
Visuospatial and Executive Function	1.04 ± 1.38	1.67 ± 1.68	4.30	<0.01
Naming Ability	1.48 ± 1.17	2.03 ± 1.04	4.93	<0.01
Attention	3.02 ± 2.16	3.93 ± 1.92	4.36	<0.01
Language Ability	0.80 ± 0.87	1.38 ± 1.05	6.35	<0.01
Abstract Thinking	0.29 ± 0.59	0.57 ± 0.78	4.34	<0.01
Delayed Recall	0.55 ± 1.15	1.12 ± 1.53	4.58	<0.01
Orientation	4.18 ± 1.81	4.93 ± 1.47	4.43	<0.01

A multiple linear regression analysis was conducted with the total MoCA score of the research group as the dependent variable. Independent variables included the research group’s gender, age, residence area, higher educational level, marital status, living arrangement, employment status, source of medical care, remission status of depression, duration of depressive episodes within five years, use of antidepressant medication, presence of other mental illnesses, presence of physical illnesses, and total scores for GHQ-12, QLQ, and PSQI from both 2015 and 2020, as well as GPS scores from 2020. The results indicated that gender, age, residence area, years of education, QLQ total score from 2015, and GHQ-12 total score from 2015 were factors affecting cognitive function in depressive disorders (*P* < 0.05). Specifically, males, individuals with better early-life quality of life, poorer early psychological conditions, and higher educational levels exhibited better cognitive function. Conversely, older age and rural residence were associated with poorer cognitive function. Upon standardizing the partial regression coefficients of each influencing factor, it was determined that years of education and age are the primary and secondary influencing factors for cognitive function. For more details, see **Table 5**.

**Table 5:** Factors influencing cognitive function in the research group: multiple linear regression.

Factors	*β*	*SE*	*B*	*t*	*P*	*VIF*
Male ^a^	1.72	0.50	0.11	3.45	<0.01	1.32
Age	-0.18	0.02	-0.29	-8.33	<0.01	1.45
Rural residence ^b^	-1.12	0.47	-0.07	-2.38	0.02	1.17
Years of education	0.92	0.07	0.49	13.69	<0.01	1.57
QLQ total score(2015)	0.06	0.03	0.08	2.28	0.02	1.48
GHQ-12 total score(2015)	0.17	0.07	0.08	2.29	0.02	1.33

R^2^ = 0.60, adjusted R^2^ = 0.58, F=37.00, P<0.01, Durbin-Watson=2.09.

a Female as a reference.

b Urban residence as a reference.

## 4 Discussion

### 4.1 Analyzing the influence of demographic and social factors on cognitive function

Current research suggests that socio-psychological factors are crucial in influencing cognitive functions. In this study, a higher level of education is identified as the primary protective factor for cognitive functions. The positive impact of educational attainment on cognitive functions has been confirmed by numerous studies. Studies consistently demonstrate a positive correlation between higher levels of education and enhanced cognitive function across various domains (22). Lenehan et al. similarly found through a review of studies that individuals with higher levels of education exhibit better cognitive function among peers (23). Higher levels of education are often associated with better cognitive reserve (24), leading to better coping with the risk of cognitive decline.

In this study, advanced age is identified as the most significant risk factor for cognitive impairment. As the body ages, cognitive functions also decline, encompassing processing speed, attention, memory, and executive functions (25). This decline may be due to the degradation of the nervous system, such as reductions in gray and white matter volume and decreases in nerve conduction speed (26). Although age is a significant factor in cognitive decline, there are substantial individual differences among the elderly, influenced by genetics, lifestyle, and educational level (27). For instance, physical exercise can mitigate or even partially reverse cognitive decline (28), with higher-educated elderly individuals exhibiting better cognitive functions (29). Thus, the cognitive functions of the elderly require more attention and can be preserved by improving lifestyle and increasing educational levels.

Other demographic factors are also closely related to the development of cognitive impairments. An epidemiological investigation revealed that unemployment, divorce, widowhood, or solitary living are associated with diminished cognitive capabilities in older adults (30), aligning with the outcomes of our research. However, our study found that employed individuals displayed lower cognitive functions, which may relate to the high proportion of the rural population in the survey. People in rural areas often engage in physically demanding jobs with high stress and low satisfaction, leading to negative emotions like tension and anxiety, which in turn may cause cognitive impairments such as decreased attention, decision-making abilities, and working memory. Zhuo et al.’s study supports this view (31). Residing in rural areas is a risk factor for cognitive function in this study, which may be associated with poorer economic conditions and cultural levels in rural areas, limited health literacy, social support, and medical resources, as well as lower life satisfaction (32). Regarding gender, this study concluded that males have better cognitive functions, whereas Brown et al. suggest a link between males and subjective cognitive decline (33). In China, women generally have lower educational levels than men, which may influence the relationship between gender and cognitive function (34), hence the study findings may be linked to this disparity.

Additionally, this study found that in the general study population, higher quality of life was significantly associated with better cognitive function. In contrast, among individuals with depressive disorders, only early-life quality of life was related to cognitive function, while the relationship between current quality of life and cognitive function was not significant. Better early-life quality of life may reflect long-term favorable social support and mental health, thus providing protective benefits to cognitive function (35). Current quality of life more reflects short-term living conditions and psychological state. Compared to the general population, individuals with depressive disorders are adversely affected by their illness, leading to poorer mental health and reduced sensitivity to external stimuli, which may diminish the short-term impact of quality of life on cognitive function. Additionally, this study found that poorer early psychological conditions were associated with better cognitive function. Individuals with poorer early psychological conditions may have adopted proactive coping strategies, which could contribute to the preservation of cognitive function later (36). Although many studies have confirmed the impact of sleep quality on cognitive functions, this study did not find a correlation between the two. Although covering all age groups, the average age of participants was 64, and nearly 70% were female. In comparison to younger and male populations, older individuals and women often experience poorer sleep quality (37, 38), which may have influenced the findings of this study.

### 4.2 Analyzing the influence of depressive disorders on cognitive functions

In this study, the cognitive functions of the depressed population were significantly lower across various domains compared to the general population, consistent with previous research findings. Research suggests that depressive symptoms predict lower episodic memory and executive function (39), and performance is significantly poorer in working memory, processing speed, delayed recall, and orientation (40, 41). Moreover, depressive symptoms increase the likelihood of declines in cognitive functions (42), and as depressive symptoms intensify, the impairment in cognitive functions worsens (43). In this study, there were no significant differences between the research group and the elevated risk group in the cognitive domains of naming ability, delayed recall ability, and orientation, suggesting potentially that the impact of depressive disorders on these areas is relatively smaller compared to other cognitive domains. Austin et al. noted that although depressive disorders affect overall cognitive function, their impact on naming ability and orientation is relatively minor (44). Snyder also found that executive functions are more significantly affected by depression compared to naming ability, delayed recall, and orientation (45). Additionally, the elevated risk group often faces more stress, adverse life events, and negative emotions, or is restricted by inherent personality traits in mitigating the impact of these emotions, leading to impairment in certain cognitive functions (6), which results in no significant differences in some cognitive domains compared to the depressed population. It is evident that although depressive disorders broadly affect cognitive functions, the extent of their impact may vary across different cognitive domains.

The research findings revealed that the cognitive function of the depression-remitted group surpassed that of the unremitted group in all domains, suggesting that with the improvement of depressive symptoms, patients may experience a certain degree of recovery in cognitive functions (46). However, in this study, depression remission was not a factor affecting cognitive function in people with depressive disorders, suggesting that the cognitive impairments caused by depressive episodes are not entirely transient. Conradi et al.’s research found that cognitive impairments generally persist even after depression remission (3). A subsequent study also unveiled that visuospatial abilities, information processing speed, and delayed memory continue to be impaired after depression remission (47). This may be due to depressive disorders leading to a reduction in neural synapses in brain structures such as the prefrontal cortex and hippocampus (48, 49), resulting in persistent cognitive impairments. Additionally, a long-term imbalance in neurotransmitters such as serotonin, dopamine, and norepinephrine may impact the recovery of cognitive functions (50).

### 4.3 Limitations

In this study, the mean age of the surveyed population was relatively high. This may be related to the high proportion of rural participants in the survey, combined with the social phenomenon of a large number of young and middle-aged individuals from rural areas in China migrating for work in recent years. This age disparity may influence the generalizability of the study’s findings. Moreover, during the 2015 epidemiological survey of mental disorders in Shandong Province, cognitive function assessments were not conducted due to considerations of workload and feasibility. Consequently, it was not possible to perform a comparative analysis of cognitive function among the three groups before and after the five-year period.

## 5 Conclusions

Individuals with depressive disorders often suffer widespread cognitive impairment, which shows some degree of recovery when depression is alleviated. Individual cognitive function is influenced by factors such as age, gender, place of residence, educational level, occupation, marital status, early psychological health, depression status, and quality of life. Therefore, when formulating corresponding treatment strategies, these factors can be comprehensively considered to develop targeted interventions that facilitate the recovery of cognitive function in individuals with depressive disorders.

## Data Availability

The original contributions presented in the study are included in the article/supplementary material. Further inquiries can be directed to the corresponding authors.
